# Modelling the social and structural determinants of tuberculosis: opportunities and challenges

**DOI:** 10.5588/ijtld.16.0906

**Published:** 2017-09-01

**Authors:** D. Pedrazzoli, D. Boccia, P. J. Dodd, K. Lönnroth, D. W. Dowdy, A. Siroka, M. E. Kimerling, R. G. White, R. M. G. J. Houben

**Affiliations:** * TB Modelling Group, TB Centre and Centre for the Mathematical Modelling of Infectious Diseases, London; † Department of Infectious Disease Epidemiology, London School of Hygiene & Tropical Medicine, London; ‡ Health Economics and Decision Science, School of Health and Related Research, University of Sheffield, Sheffield, UK; § World Health Organization, Global Tuberculosis Programme, Geneva, Switzerland; ¶ Department of Public Health Sciences, Karolinska Institutet, Stockholm, Sweden; # Department of Epidemiology, Johns Hopkins Bloomberg School of Public Health, Baltimore, Maryland, USA; ** KNCV, Tuberculosis Foundation, The Hague, The Netherlands

**Keywords:** mathematical modelling, tuberculosis, social determinants

## Abstract

**INTRODUCTION:**

Despite the close link between tuberculosis (TB) and poverty, most mathematical models of TB have not addressed underlying social and structural determinants.

**OBJECTIVE:**

To review studies employing mathematical modelling to evaluate the epidemiological impact of the structural determinants of TB.

**METHODS:**

We systematically searched PubMed and personal libraries to identify eligible articles. We extracted data on the modelling techniques employed, research question, types of structural determinants modelled and setting.

**RESULTS:**

From 232 records identified, we included eight articles published between 2008 and 2015; six employed population-based dynamic TB transmission models and two non-dynamic analytic models. Seven studies focused on proximal TB determinants (four on nutritional status, one on wealth, one on indoor air pollution, and one examined overcrowding, socioeconomic and nutritional status), and one focused on macro-economic influences.

**CONCLUSIONS:**

Few modelling studies have attempted to evaluate structural determinants of TB, resulting in key knowledge gaps. Despite the challenges of modelling such a complex system, models must broaden their scope to remain useful for policy making. Given the intersectoral nature of the interrelations between structural determinants and TB outcomes, this work will require multidisciplinary collaborations. A useful starting point would be to focus on developing relatively simple models that can strengthen our knowledge regarding the potential effect of the structural determinants on TB outcomes.

## Abstract (FR)

**INTRODUCTION:**

En dépit du lien étroit entre tuberculose (TB) et la pauvreté, la majorité des modèles mathématiques de TB n'ont pas examiné les déterminants sous-jacents sociaux et structurels (SD). Dans cet article, nous revoyons lesétudes employant une modélisation mathématique afin d'évaluer l'impact épidémiologique des SD de la TB.

**MÉTHODE:**

Nous avons fait une recherche systématique sur PubMed et dans des bibliothèques personnelles pour identifier les articles éligibles. Nous avons extrait des données relatives aux techniques de modélisation employées, aux sujets de recherche, le type de SD modélisés et le contexte.

**RÉSULTATS:**

A partir de 232 documents identifiés, nous avons inclus huit articles publiés entre 2008 et 2015 ; six ont employé des modèles dynamiques basés en population de la transmission de la TB. Sept études se sont focalisées sur les déterminants proximaux de la TB (quatre sur le statut nutritionnel, une sur le niveau économique, une sur la pollution de l'air intérieur et une a examiné la surpopulation et le statut socioéconomique et nutritionnel) et une s'est concentrée sur les influences macroéconomiques.

**CONCLUSION:**

Peu d'études de modélisation ont tenté d'évaluer les SD de la TB, ce qui a abouti à des lacunes majeures en termes de connaissances. En dépit des défis de la modélisation d'un système aussi complexe, les modèles doivent élargir leur portée pour rester utiles aux décisions de politique. Etant donné la nature intersectorielle des interrelations entre SD et évolution de la TB, ce travail va requérir une collaboration multidisciplinaire. Un point de départ utile consisterait à se concentrer sur l'élaboration de modèles relativement simples qui peuvent renforcer nos connaissances relatives à l'effet potentiel des SD sur l'évolution de la TB.

## Abstract (SP)

**MARCO DE REFERENCIA:**

Pese al vínculo estrecho entre la tuberculosis (TB) y la pobreza, la mayor parte de los modelos matemáticos de la TB no aborda los determinantes sociales y estructurales (SD) subyacentes. En el presente estudio se revisaron los artículos que empleaban modelizaciones matemáticas con el propósito de evaluar la repercusión de los SD en las características epidemiológicas de la TB.

**MÉTODOS:**

Se llevó a cabo una búsqueda sistemática en la base de datos PubMed y las bibliotecas personales con el fin de localizar publicaciones idóneas. Se extrajeron datos sobre las técnicas de modelización utilizadas, la pregunta de la investigación, el tipo de SD modelizados y el entorno.

**RESULTADOS:**

De los 232 documentos localizados, se incluyeron 8 artículos publicados del 2008 al 2015; en 6 publicaciones se utilizaban modelos poblacionales dinámicos de transmisión de la TB y 2 utilizaban modelos analíticos no dinámicos. Siete estudios se centraban en los determinantes próximos de la TB (4 en el estado nutricional, 1 en la riqueza, 1 en la contaminación del aire de interiores y otro examinaba el hacinamiento, la situación socioeconómica y el estado nutricional) y un estudio abordaba las influencias macroeconómicas.

**CONCLUSIÓN:**

Pocos estudios de modelización han tratado de evaluar los SD de la TB, por lo cual existen lagunas importantes en los conocimientos. Pese a las dificultades que plantea la modelización de un sistema tan complejo, los modelos deben ampliar su ámbito, con el objeto de contribuir a la formulación de políticas. Dado el carácter intersectorial de las interrelaciones entre los factores sociales y estructurales y los desenlaces de la TB, este trabajo exige colaboraciones multidisciplinarias. Un punto de partidaútil sería centrarse en la elaboración de modelos relativamente sencillos que amplíen los conocimientos sobre el posible efecto de los SD en los desenlaces de la TB.

TUBERCULOSIS (TB) is widely recognised as a disease of poverty,^1–3^ with disproportionate disease burden falling on the poorest in society and the most vulnerable communities. The need to design and implement comprehensive strategies to achieve TB elimination through universal health coverage and interventions to address the underlying social determinants of TB is a key element of the World Health Organization's (WHO's) End TB strategy for 2015–2035.^4^,^5^

The targets and indicators of this new TB action framework are anchored in the 17 Sustainable Development Goals (SDGs) adopted by the United Nations and which mark the global development agenda that began on 1 January 2016. By placing their emphasis on the interdependence and synergies between socio-economic development and health,^6^ these offer unique entry points for addressing the social determinants (SDs) of TB.

In the present article, we follow the definition of SDs of health of the WHO Commission on Social Determinants of Health:^7^ ‘The structural determinants of TB are those conditions that generate or reinforce social stratification (e.g., socio-economic inequalities, population growth, urbanisation), and therefore give rise to an unequal distribution of key social determinants of TB epidemiology, such as poor housing, poverty and malnutrition, which in turn influence exposure to risk, vulnerability and ability to recover after developing the disease.’^8^ These definitions are shown in Table 1.

**Table 1:**
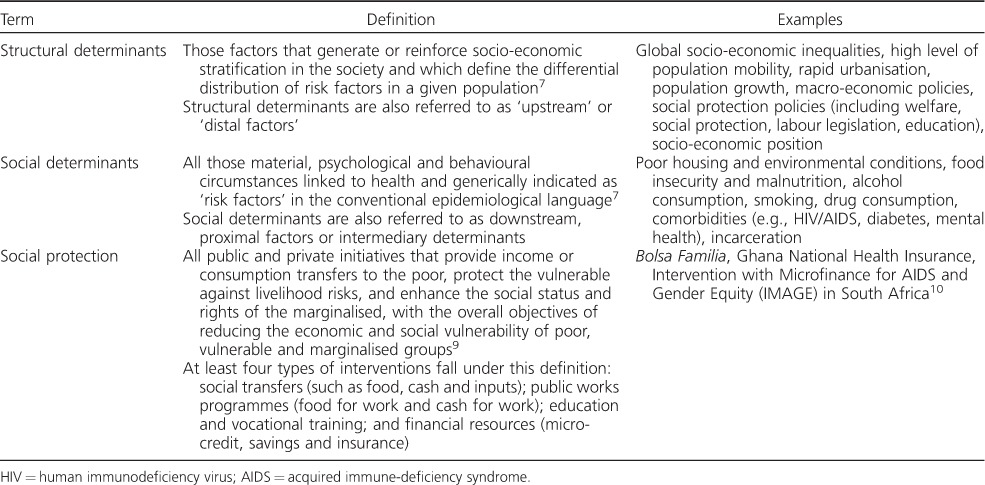
Structural and social determinants, and social protection: definitions and examples

Quantitative analytical tools such as mathematical modelling can play an important role in informing the End TB Strategy, evaluating the impact of novel poverty-reduction interventions nested in its vision (including in combination with existing biomedical tools), and exploring the contribution of socioeconomic drivers to the epidemic. However, to do so, TB models will inevitably need to expand their focus beyond diagnosis and treatment to incorporate SDs, but the potential of modelling as well as its main limitations in supporting this research agenda remain unclear.

In the present paper, we report findings from a systematic review of the literature carried out with the aim to provide an overview of the current state of knowledge in the mathematical modelling of SDs of TB. We then go on to discuss key methodological challenges and gaps in empirical evidence that existing mathematical models need to overcome to be able to incorporate SDs to remain relevant to policy-making.

## METHODS

### Search strategy and selection criteria

For the purposes of this review, ‘mathematical model’ was defined based on that envisaged by Garnett et al. as a mechanistic representation of how disease burden is established. This includes both dynamic transmission and decision (non-dynamic) analytic models. We searched PubMed for any relevant article on modelling and the socio-economic determinants of TB (e.g., nutrition, crowding, poverty).^11^ The full search string is included in Table 2. Titles and abstracts were screened for eligibility. Articles were eligible for full-text review if they were written in English (due to limited resources), the target population was human individuals and mathematical modelling assessed the epidemiological impact of the SDs of TB.

**Table 2:**
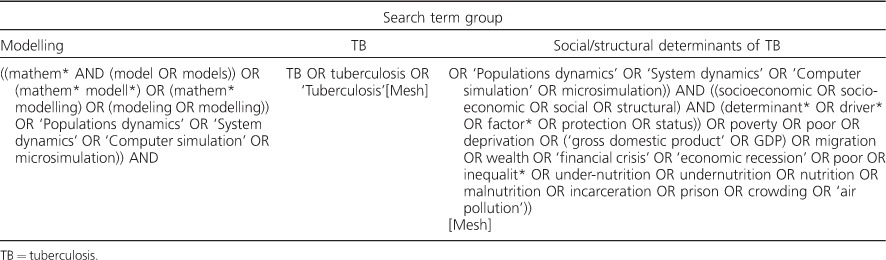
Full search string for literature review in the PubMed database

We excluded systematic reviews, epidemiological studies that did not use mathematical modelling techniques and ecological analyses looking at the SDs of TB. The search focused on socio-economic factors (i.e., the intervention or exposure involving a socioeconomic factor), and excluded studies focusing only on diabetes mellitus (DM), the human immunodeficiency virus (HIV) and behavioural risk factors such as alcohol consumption and smoking unless their association with socio-economic factors were also considered. We applied no restrictions as to the year.

Additional relevant articles were identified in the authors' personal libraries and are included in the review. DP selected the articles with support from RMGJH, DB and KL; data were extracted by DP and RMGJH.

### Data abstraction and synthesis

**Figure 1:**
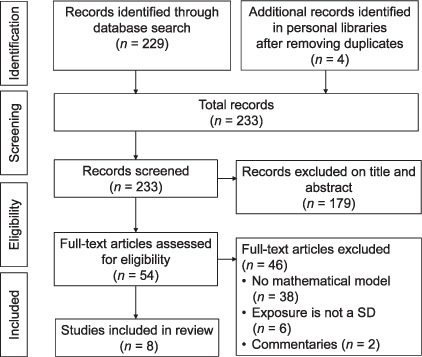
Flowchart for article selection for the systematic review. SD = social determinant.

Figure 1 presents the details of the selection process. The aim of the study, first author and publication dates, type and feature of the model, the socioeconomic factor, the setting and the main findings were extracted into a pre-designed form. We focused on a qualitative synthesis of the methods employed in the articles we identified.

## RESULTS

A total of 229 unique records were found in the literature search, and four additional articles were added from the authors' personal libraries. Of these, 54 underwent full-text evaluation. After full-text screening, we included eight articles published between 2008 and 2015, with four articles published in 2015 only. Table 3 gives the main features of the selected studies.^12–19^

**Table 3:**
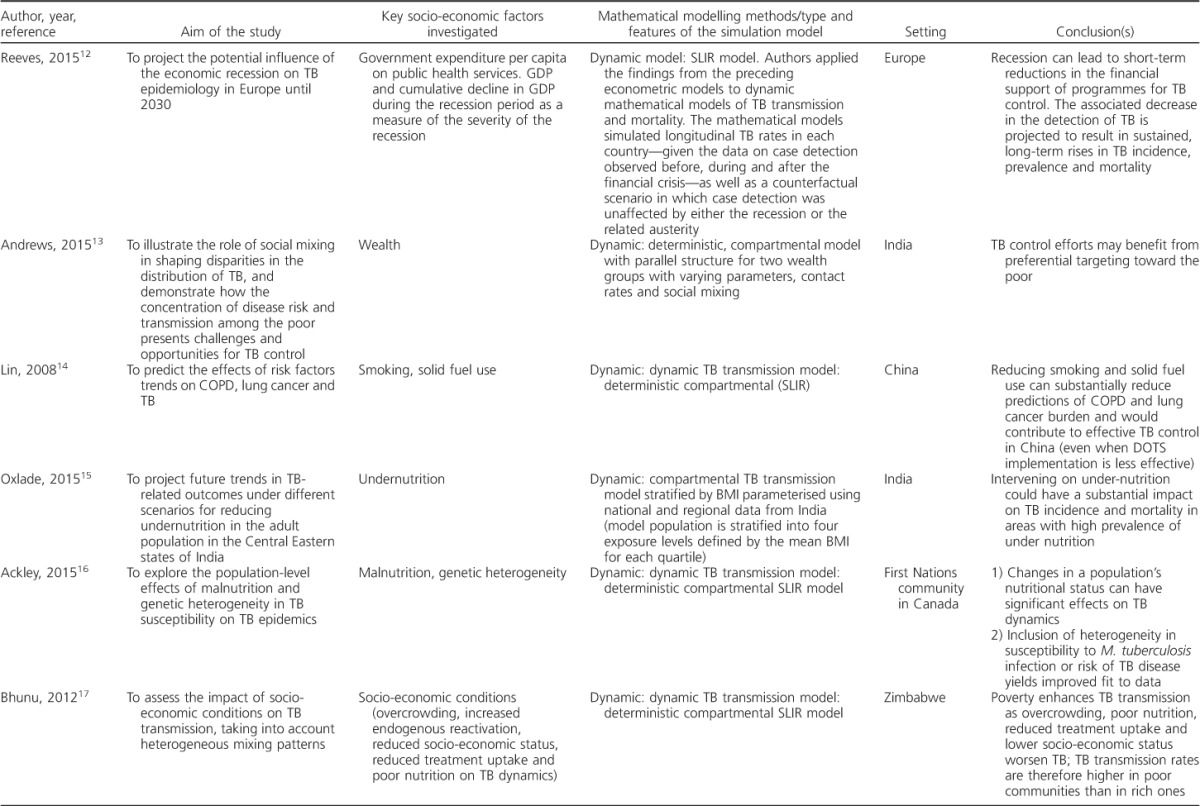
Summary of studies identified in the systematic review

**Table 3:**
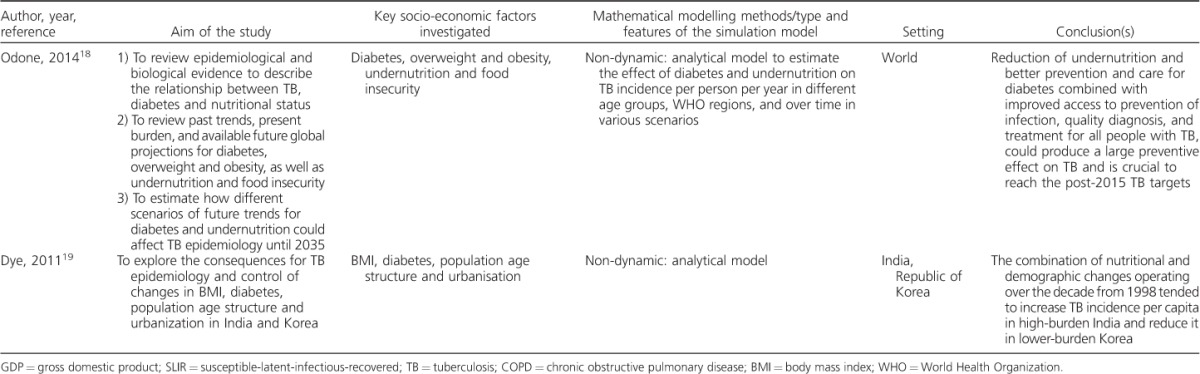
(continued)

### Socio-economic factors investigated

The study by Reeves et al. was the only article that looked at the impact of distal determinants, i.e., government expenditure per capita on public health services, gross domestic product (GDP) and cumulative decline in GDP, as a measure of the severity of an economic recession on TB control.^12^ The remainder modelled proximal TB determinants: four focused on nutritional status (body mass index [BMI] and undernutrition),^14^,^16^,^18^,^19^ one on wealth,^15^ one on smoking and indoor air pollution,^13^ and one on nutritional status, overcrowding and socio-economic status.^17^ All studies looked at one factor at a time, with the exception of the study by Dye et al.,^19^ which also explored the combined effect of nutritional status and demographic changes, including age structure and urbanisation, on TB incidence.

### Modelling methods, structure and parameters

Compartmental population-based dynamic TB transmission models were the most common simulation approach employed in the selected articles (6/8, 75%); two studies used non-dynamic analytical models and both investigated the effect of both DM and nutritional status on TB epidemics. Most studies included a conceptual framework to illustrate the mechanics of the models and the hypotheses behind their research questions.

Transmission models employed standard SLIR (susceptible-latent-infectious-recovered) models that were adapted to explore the research question set in each study: the model by Oxlade et al., for example, was stratified by levels of undernutrition by wealth quartile.^15^ Andrews et al. implemented a parallel structure for two wealth groups to a standard TB model to explore the benefit of assortative mixing to interventions targeting the poor, highlighting the potential importance of including mixing parameters in TB models even if data are currently not available to inform these models.^13^

With regard to model parameters, Ackley et al. explored changes in differences in susceptibility to infection and progression to disease in hypothetical scenarios.^16^ Different levels of BMI drove changes in reactivation and progression parameters in the model used by Oxlade et al.^15^ The study by Reeves et al. used an econometric analysis to estimate changes in relevant model parameters controlling case detection.^12^ Bhunu et al. divided the population into ‘rich’ and ‘poor’ communities, and compared the reproduction numbers for these two strata (Appendix Table A).^17^^*^

Data on the different exposures were mainly drawn from the literature,^16^,^18^ national population-based surveys^13–15^,^19^ or publicly available databases.^12^ Very few data employed in these studies were local or regional. The majority of the studies were calibrated to TB data (e.g., incidence trends or point estimates) from WHO estimates.

### Key findings of the modelling studies

The studies in our review support the notion that TB control is linked to and would benefit from action on TB social determinants. Reeves et al. found that a decrease in funding to control TB due to an economic recession (distal factor) can lead to a decline in TB case detection, and consequently to higher TB rates.^12^ Lin et al. showed that interventions on smoking and indoor air pollution (proximal factors) can accelerate TB decline.^20^ The studies that focused on nutritional status (proximal factor) found that reducing under-nutrition would substantially reduce TB incidence. Andrews et al. showed that preferential targeting of the poor can benefit TB control (wealth as a proximal factor).^13^ From the analysis of reproduction numbers for the poor and rich communities, Bhunu et al. found that overcrowding, poor nutrition, lower socioeconomic status (proximal factors) and reduced TB treatment uptake worsened TB transmission.^17^ Finally, the study by Dye et al. concluded that a combination of nutritional and demographic changes (proximal factors) operating over the decade from 1998 tended to increase TB incidence per capita in high-burden India but reduce it in lower-burden Korea.^19^

## DISCUSSION

This review has highlighted the paucity of mathematical modelling studies looking at the effects of socio-economic factors on TB pathogenesis and epidemiology, but has also shown that, although fairly recent, work in this field seems to be growing as the number of articles published has increased from 2011 onwards. This is possibly a reflection of changing policy priorities that are now part of the End TB Strategy.

Our findings point to the need, at this stage, to develop relatively simple models that improve and expand the current body of work to incorporate available evidence and strengthen our knowledge of the potential effect of SDs on TB outcomes. For instance, most models focused on one or two factors only, and those that considered two factors did not account for possible interactions between them. It is to be noted that most mathematical modelling studies focussed on assessing the effect of nutritional status and changes in BMI on TB epidemiology. This is not surprising, as undernutrition has long been acknowledged as a key socially determined TB risk factor. We found no modelling work looking at the impact of improved socio-economic macro-indicators on TB outcomes, or of social protection interventions targeting TB patients and their households. With respect to proximal risk factors, only one model assessed the effect of crowding on TB epidemiology, possibly a reflection of the fact that data on crowding and TB are not rich enough to unpick causality for a model.

### Challenges in translating from determinant to model

**Figure 2:**
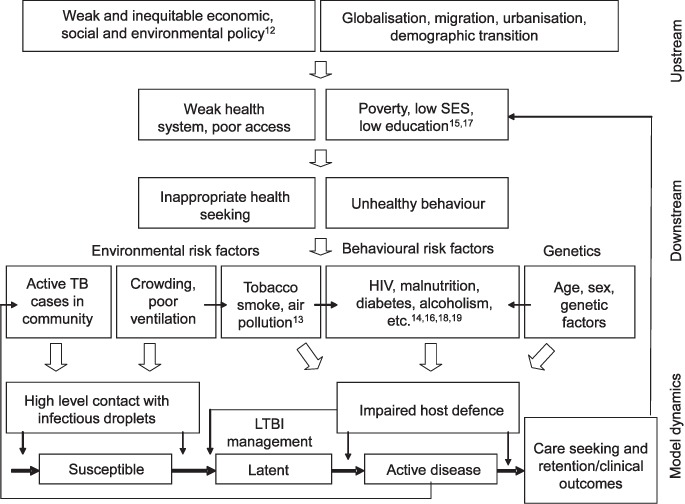
Framework for proximate risk factors, upstream determinants and TB mechanics. Source: Adapted from Lönnroth et al.^2^ This framework provides an example of the complexity when considering SDs in TB models: it illustrates the complicated cascade of parameters from distal to downstream determinants affecting the development of disease, and care and prevention. SD = structural determinants; SES = socio-economic status; HIV = human immunodeficiency virus; LTBI = latent tuberculous infection.

The narrow focus of past global health and development policies and TB control strategies only partly explains why TB modelling has so far shown some reluctance to include SDs. This has also been due to the real and perceived weaknesses in the empirical evidence needed to populate models and quantify the pathways from socio-economic factors to changes in the natural history of TB in a population. Figure 2 provides a conceptual framework that outlines how distal/structural determinants (such as macro-economic policies) work through a potential array of more proximal determinants (e.g., crowding and nutrition), which in turn affects the dynamics of a standard mechanistic TB model at multiple points,^21^,^22^ such as the intensity of transmission (through crowding) or the rate of progression after recent and/or latent infection (e.g., through nutrition) (Figure 2).

While there are some data to inform parts of, for example, the pathway from macro-economic policies (e.g., GDP) to TB incidence,^12^ our ability to quantify the exact relationship of each step remains limited. However, it should be noted that the same limitations apply to current TB models, ranging from capturing the impact of HIV or, when models look to evaluate the potential impact of interventions, including current approaches to improving case detection and reducing patient delay, or future hypothetical tools.^23^,^24^

When translating the effect of changing a socioeconomic determinant into a mechanistic model, it does not suffice to have an estimate of the magnitude of the effect (see examples in Table 4).^20^,^25–34^ One needs to know, or make assumptions about, the model parameters that should be changed to achieve the estimated impact. As shown in Figure 2, changes in disease risk may be due to influences at one or several of the stages on the pathway between exposure and disease that are captured by transmission models. As direct evidence is often still lacking, this means that choices need to be made based on the likely biological mechanism.

**Table 4:**
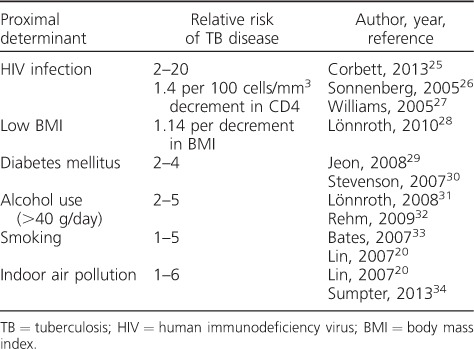
Known relationships between proximal determinants and the risk of developing TB disease

The range of these potential model parameters includes those that directly capture the intensity of transmission, e.g., social mixing or crowding in households, but also parameters guiding progression to disease after infection, which can be affected, for example, by nutritional status. It is also plausible that different paths to progression (primary, reactivation, reinfection) are affected at different stages of the pathway. In addition, any interventions that reduce barriers to care and treatment completion will change model parameters capturing the time to diagnosis as well as retention in care (e.g., alcohol and drug abuse).

In addition to effects on incidence, SDs may alter the natural history of disease (e.g., reduced infectiousness and disease duration in people living with HIV) or disease outcomes (e.g., HIV, undernutrition, DM and smoking). Clustering of these risk factors for behavioural or biological reasons requires an understanding of their interactions, and further increases the level of knowledge required. Finally, separating out composite phenomenological quantities into their mechanistic components may also improve transferability between settings if the data needed to quantify how these components differ are available.

## CONCLUSIONS AND RECOMMENDATIONS

Mathematical modelling is a powerful and flexible tool to inform policy discussions and estimate the potential impact of various interventions relative to one another.^11^ However, to be useful, models need to be able to reflect the relevant aspects of the epidemic and address the questions faced by policy makers. In the SDGs and End TB Strategy era, this means that mathematical models of TB must translate the impact of socio-economic determinants into their mechanistic components. As a starting point, the TB modelling community should use the existing scientific evidence to construct relatively simple mechanistic models that add to our understanding of the effect of SDs on TB, and help improve specific policy decisions.

As shown in this article, there exists a scarcity of TB models that include SDs, but also a small but increasing body of work that has explored initial ideas. Some modelling of proximal risk factors and related public health interventions has been done but, for example, this has never moved upstream. TB models can leverage the existing data, and highlight the value of collecting those that are missing, such as the exact link between changes in nutritional status and changes in progression to disease, or the relationship between transmission intensity and living environments (e.g., urban slums compared with rural settings).

To further our knowledge, projects are urgently needed that advance the field while avoiding the pitfall of developing overly complex models that include population or pathway structures not adequately supported by empirical evidence or fully understood. In addition, the complexity of the pathways involved and the multisectoral nature of new approaches to end TB evidently require collaborations from different disciplines, including social scientists, epidemiologists, economists, policy makers as well as mathematical modellers.^13^ While recognising the importance of such projects but at the same time the struggle to identify suitable funding opportunities for such cross-disciplinary collaborative work, the TB Modelling and Analysis Consortium organised a meeting at the end of 2015 to discuss existing experiences and the potential path forward. A range of projects was developed that would both advance the field and be feasible, given current data.^35^ Two of these projects have been funded, preliminary results were produced at the end of 2016 and publications are under review: an interdisciplinary project looking at how social protection interventions can accelerate TB elimination (the Social Protection to Enhance the Control of TB Consortium, S-PROTECT), and a project assessing the relative contribution of TB programme (DOTS) expansion and improvements in socio-economic indicators on TB epidemiology in China.

In this article, we highlighted that the literature on mathematical modelling of social determinants of TB remains limited. We argue that to maintain its key role in policy discussions in the era of the SDGs and End TB Strategy, the TB modelling community needs to embrace the technical challenges to adequately represent the interplay between TB and its socioeconomic drivers. While some work is underway, more funding, data and capacity are urgently needed to ensure TB modelling remains a useful tool for the ultimate goal of TB elimination.

## Figures and Tables

**Table A:**
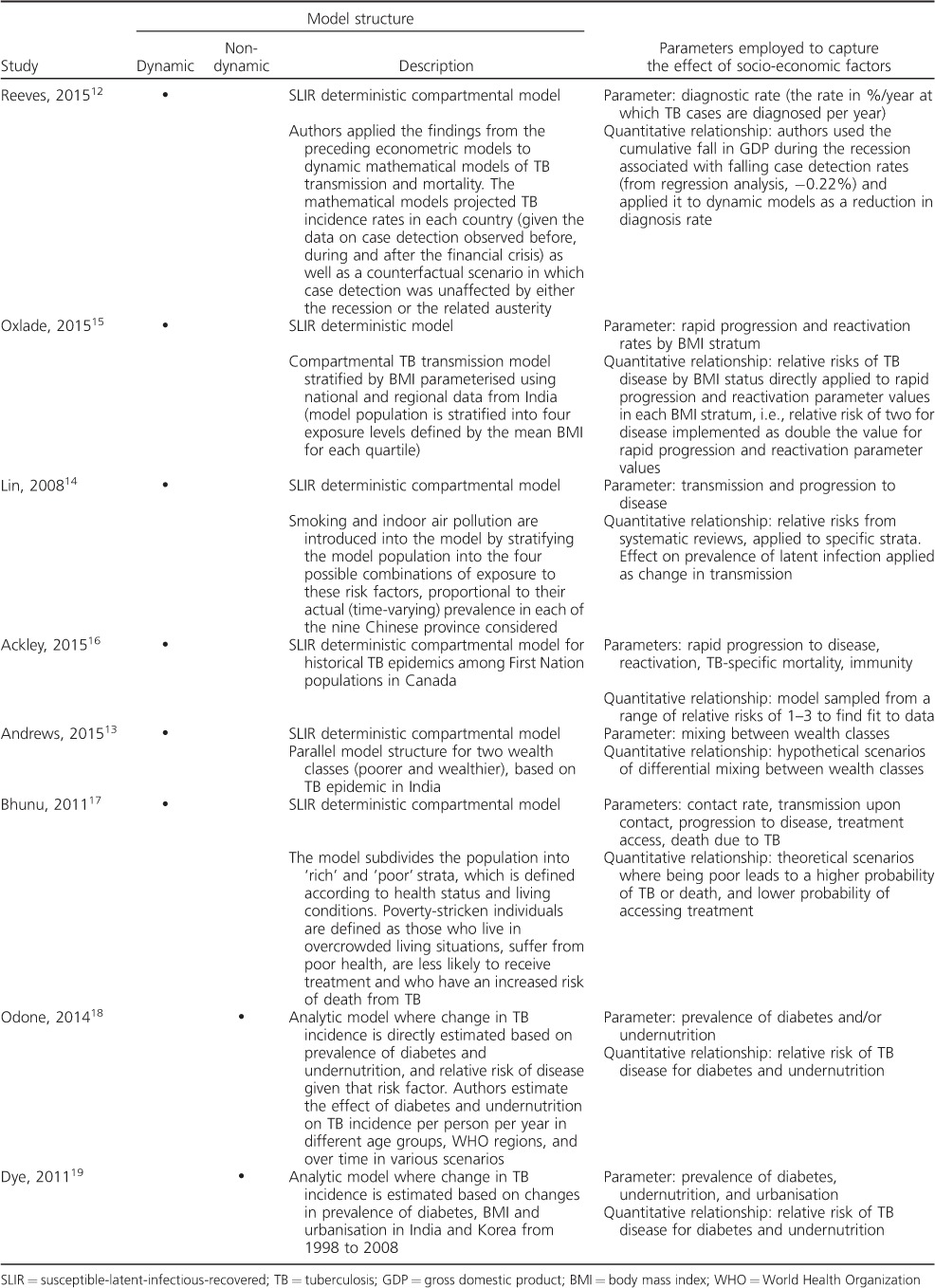
Summary of model structure and parameters employed in the studies included in the review

## Footnotes

* The appendix is available in the online version of this article, at http://www.ingentaconnect.com/content/iuatld/ijtld/2017/00000021/00000009/art00005
